# Effects of *Bacillus licheniformis* on the Growth Performance, Antioxidant Capacity, Ileal Morphology, Intestinal Short Chain Fatty Acids, and Colonic Microflora in Piglets Challenged with Lipopolysaccharide

**DOI:** 10.3390/ani13132172

**Published:** 2023-07-01

**Authors:** Guangtian Cao, Shenglan Yang, Huixian Wang, Ruiqiang Zhang, Yanping Wu, Jinsong Liu, Kaifan Qiu, Yingkun Dong, Min Yue

**Affiliations:** 1 College of Animal Science, Zhejiang University, Hangzhou 310058, China; 15a1903025@cjlu.edu.cn; 2 College of Standardisation, China Jiliang Universtiy, Hangzhou 310058, China; aoqin1314@163.com (K.Q.); ddyykkqvq@163.com (Y.D.); 3 College of Animal Science and Technology, Zhejiang A & F University, Hangzhou 311300, China; yangshenglan7@163.com (S.Y.); 18864833071@163.com (H.W.); zrq1034@163.com (R.Z.); ypwu0902@163.com (Y.W.); 4 Zhejiang Huijia Biotechnology Co., Ltd., Huzhou 313307, China; 13906510961@163.com

**Keywords:** *Bacillus licheniformis*, colonic microflora, growth performance, lipopolysaccharide challenging, piglets

## Abstract

**Simple Summary:**

Weaning is a critical period in sow production and induces oxidative damage, which is closely associated with diarrhea, intestinal metabolic disorders, and infections. Probiotics as substitutes for antibiotics play a beneficial role in decreasing diarrhea and improving the growth of weaning piglets. The intraperitoneal injection of lipopolysaccharides (LPS) has been used for inducing oxidative damage and intestinal injury in piglets. In the present study, we investigated the effects of dietary *Bacillus licheniformis* (BL) on production performance, serum antioxidant capacity, ileal morphology, intestinal short-chain fatty acids (SCFAs), and colonic microflora in piglets challenged with LPS. The results showed that BL supplementation improved growth performance, increased serum catalase activity, decreased malondialdehyde concentration, and increased colonic major SCFAs. Moreover, metagenome analysis showed that *Prevotella* species were the predominant bacteria in BL- and CBL-treated piglets. In summary, a basal diet supplemented with 10^10^ CFU BL improved production performance, serum antioxidative capacity, and ileal morphology, and modulated the colonic microflora. This experiment offers theoretical support for dietary probiotic utilization in piglets to decrease weaning stress in the sow industry.

**Abstract:**

The aim of the present study was to investigate the effects of *Bacillus licheniformis* (BL) on the growth performance, antioxidant capacity, ileal morphology, intestinal fecal short-chain fatty acids, and microflora of weaned piglets challenged with lipopolysaccharide (LPS). Piglets were assigned into three groups: basal diet (Con), a basal diet with added 10^9^ CFU *B. licheniformis*/kg (BLl), and a basal diet with added 10^10^ CFU *B. licheniformis*/kg (BLh). On day 28, BLh piglets were intraperitoneally injected with LPS (CBL) and sterilized saline water (BL), Con piglets were injected with LPS (LPS) and sterilized saline water (Con), with the injections being administered for three consecutive days. The average daily gain significantly increased from day 1 to day 28 and the feed: gain ratio decreased with BL supplementation compared with the Con group. Supplementation with BLl and BLh reduced the diarrhea rate in piglets. Serum catalase activity increased and malondialdehyde concentration decreased in the CBL treatment group compared with the LPS treatment group. Both BL and CBL treatments increased the ileal villus length/crypt depth ratio compared with Con and LPS treatments. BL administration significantly increased colonic propionic and isobutyric acid concentrations compared with Con treatment. Both BL and CBL piglets had significantly increased fecal acetic, propionic, and butyric acid levels compared with LPS piglets. Analysis of the colonic microbial metagenome showed that *Prevotella* species were the predominant bacteria in piglets treated with BL and CBL. The CBL-treated piglets had higher scores for lysine biosynthesis, arginine biosynthesis, sulfur relay system, and histidine metabolism. BL-treated piglets had higher scores for glycosaminoglycan biosynthesis-keratan sulfate, oxidative phosphorylation, and pyruvate and carbon metabolism.

## 1. Introduction

Weaning has been a critical period in sow production for decades and has often been the focus of research, as it is often associated with diarrhea, intestinal metabolic disorders, infections, and even death in piglets [1,2]. Weaning results in the destruction of the host’s antioxidant capacity, leading to oxidative stress injury [3]. Antibiotics have been utilized to defend against diarrhea and improve the growth performance of weaning piglets for a long time; however, their negative effects have meant their use is no longer favored [2]. Several studies have explored the replacement of antibiotics with probiotics, plant extracts, acidifiers, essential oils, antibacterial peptides, and other substances. Among these, *Bacillus* sp. is regarded as a promising dietary probiotic owing to its inherent ability and stress tolerance [4]. Previous studies have confirmed that *Bacillus* sp. probiotics increase digestive enzyme activity and enhance intestinal integrity and immune function, thereby improving the growth performance of swine [5,6]. *Bacillus licheniformis* (BL) is an aerobic probiotic that can degrade, absorb, and utilize nutrients, thereby restraining the growth of harmful bacteria and promoting intestinal health [7,8]. In addition, *B. licheniformis* strains have been consumed by humans for many years to stimulate the immune system [6].

The European Food Safety Authority has classified *Bacillus licheniformis* as a dietary supplement safe for use in animal production [9] and it can be effectively used as a substitute for antibiotics. Moreover, studies have revealed that supplementation with *B. licheniformis* improves growth performance and modulates digestive microflora [8,10]. Studies have also confirmed an improvement in the antioxidant capacity induced by dietary *B. licheniformis* in pigs and poultry [11,12,13]. *B. licheniformis* can play a positive role in defending against necrotic enteritis by modulating the intestinal microflora. Lipopolysaccharides (LPS) have been used to induce oxidative stress in experimental animals [14]. In weaned piglets, LPS challenge induces damage to intestinal morphology and initiates the inflammatory process [15]. Hence, intraperitoneal injection of LPS has been widely used to induce oxidative damage and intestinal injury in piglets. This trial was conducted to investigate the influence of BL administration on growth performance, antioxidative capacity, ileal morphology, intestinal short-chain fatty acids (SCFAs), and colonic microbial structure using metagenome sequencing in LPS-challenged piglets. In addition, this study was also designed to investigate the ideal dosage of BL for piglets’ growth stage, taking into account our pilot study and other studies [13].

## 2. Material and Methods

### 2.1. Animal Treatment and Designation

The experiments were conducted strictly under the Animal Management Rules of the Ministry of Health of the People’s Republic of China, and the procedures were approved by the Animal Care and Use Committee of Zhejiang University. A total of 216 weaning piglets (Duroc × Landrace × Large, half male and half female, 21 ± 1 days old) were purchased from a local farm (Anji Zhengxin Farming, Anji, China), and were derived from one batch. Piglets were randomly assigned into three treatment groups, with an initial body weight (BW) of 7.56 ± 0.32 kg, with 6 replicates per group and 12 piglets per replicate. Throughout the trial, the piglets were fed a basal diet (Con), a basal diet with added 10^9^ CFU *B. licheniformis*/kg (BLl), and a basal diet with added 10^10^ CFU *B. licheniformis*/kg (BLh). The *B. licheniformis* strain was preserved at the China General Microbiological Culture Collection Center (CGMCC, number CGMCC 23776). The piglets were free to access food and water throughout the 28-day trial. On day 28, one piglet was selected per replicate, and the 24 piglets were housed individually for the LPS challenge trial. Six piglets from the BLh treatment group were selected and intraperitoneally injected with 1 mg LPS (CBL) and another six piglets from the BLh treatment group were injected with the same volume of sterilized saline water (BL). Six piglets from the Con treatment group were injected with LPS (LPS) and another six piglets from the Con treatment group were injected with the same volume of sterilized saline water (Con), with the injections being administered for three consecutive days. LPS derived from Escherichia coli O55:B5 was purchased from Sigma Aldrich Co. (Shanghai, China). The room sanitation control and immunization schedules were consistent with those of normal management. The composition and nutritional content of the basal diet, in line with the National Research Council 2012, are listed in Table 1. The basal diet’s nutritional value was measured using the association of official analytical chemists’ procedures, which were similar to our previous study, with minor modifications [2].

Six hours after the final LPS injection, all 24 piglets from the Con, BL LPS, and CBL groups were sacrificed for sample collection. Blood was collected from the front cavity vein using an aseptic needle and placed in coagulation tubes. After blood collection, the piglets were dissected to collect samples. Serum was collected via centrifugation and stored at −20 °C for further antioxidant capacity and immune parameter analyses. Approximately 2 cm distal sections of the ileum were obtained and reserved for hematoxylin and eosin (HE) staining and analysis under scanning electron microscopy (SEM). The ileal samples were preserved in 4% formaldehyde for HE staining. The ileal samples for SEM were fixed in 3% glutaraldehyde and dehydrated using gradient ethanol concentrations. Fecal and colonic samples were stored in an aseptic cryopreservation tube at −80 °C for short-chain fatty acid (SCFA) measurement and metagenome sequencing.

### 2.2. Growth Performance

On days 14 and 28, piglets were weighed to calculate their body weight (BW) and average daily gain (ADG). Feed intake per group and diarrhea incidences were recorded daily to calculate the feed: gain (F: G) ratio and the diarrhea rate. The diarrhea rate was calculated using the formula: diarrhea rate (%) = [(number of pigs with diarrhea × diarrhea days)/(number of pigs × total observed days)] × 100.

### 2.3. Antioxidant Parameters

Serum antioxidant indices, including superoxide dismutase (SOD), malondialdehyde (MDA), glutathione peroxidase (GSH-Px), and catalase (CAT) were detected using commercial kits (Nanjing Jiancheng Institute of Biotechnology, Nanjing, China), with the detection progress measured following the manufacturers’ instructions.

### 2.4. Ileal Morphology Detection

Hematoxylin and eosin staining and SEM imaging were performed as described in our previous study [2]. Briefly, samples were embedded in Epon-Araldite; meanwhile, Leica A-1170 (Leica, Wetzlar, Germany) was used to cut slices. Uranium acetate-lead citrate was used for staining. A transmission electron microscope (H-7650; Hitachi, Tokyo, Japan) was used to obtain ultrastructural images.

### 2.5. Detection of Fecal and Colonic SCFAs

As in our previous study [2], the colonic and fecal concentrations of SCFAs (acetate, propionate, isobutyrate, butyrate, isovalerate, and valerate) were measured using Headspace Sampler Gas Chromatography (Agilent Technologies, Santa Clara, CA, USA). A 1 g sample was diluted with 4 mL of sterile water and centrifuged at 10,000× *g* and 4 °C for 15 min. The obtained supernatant was mixed with metaphosphoric acid (1:4, *m*/*v*). Finally, the supernatant was injected into the Agilent Technologies 6890N Network System (Agilent Technologies).

### 2.6. Metagenome Sequencing

Metagenome sequencing and analysis of colonic contents (a total of 9 samples, with samples from 2 piglets combined into one biological sample per treatment) were performed on the OE Biotech Co., Ltd. (Shanghai, China) platform using TruSeq Nano DNA LT Sample Preparation Kit (Illumina, Carlsbad, CA, USA) for genomic DNA extraction. The DNA library preparation workflow was fragmentation, cleaning up, end repair, 3′ ends adenylation, adapter ligation, and enrichment of DNA fragments. Other major kits used included the Agencourt AMPure XP (Beckman Coulter, Brea, CA, USA) and the KAPA Library Quantification Kits (Boston, MA, USA). Raw data were trimmed and filtered using Trimmomatic (v0.36), and valid reads were aligned using post-filtered paired-end reads. The non-redundant protein sequence database (NR), Kyoto Encyclopedia of Genes and Genomes (KEGG), and clusters of orthologous groups were used to annotate the representative gene set sequences. The taxonomy of the obtained species was derived from the NR Library database, and species abundance was measured based on the abundance of the corresponding genes. The abundance profiles were calculated at the domain, kingdom, phylum, class, order, family, genus, and species levels.

### 2.7. Statistical Analyses

The replicate was considered as the experimental unit. Statistical analyses were calculated using IBM SPSS Statistics software (version 26.0, IBM Corp., New York, NY, USA), with analysis of variance (ANOVA) and Duncan’s multiple range test. Data were presented as the mean ± standard error of the mean. Figures and images were generated using GraphPad Prism 8.0 (GraphPad Prism Inc., San Diego, CA, USA). Differences within different groups (*p* < 0.05) were considered statistically significant at 5%.

The functional abundance spectrum of the metagenome sequences was analyzed using R software (v3.2.0). Principal component analysis (PCA) and principal coordinate analysis (PCoA) were also conducted. The R package was then used to analyze significant differences between different groups using ANOVA/Kruskal Wallis/T test/Wilcoxon statistical test. The linear discriminant analysis effect size (LEfSe) method was used to compare the taxonomy abundance spectrum or functional abundance spectrum (https://bioconductor.org/packages/release/bioc/html/lefser.html, accessed on 15 January 2023), with the linear discriminant analysis (LDA) score set at 2.0.

## 3. Results

### 3.1. Growth Performance

**Figure 1:**
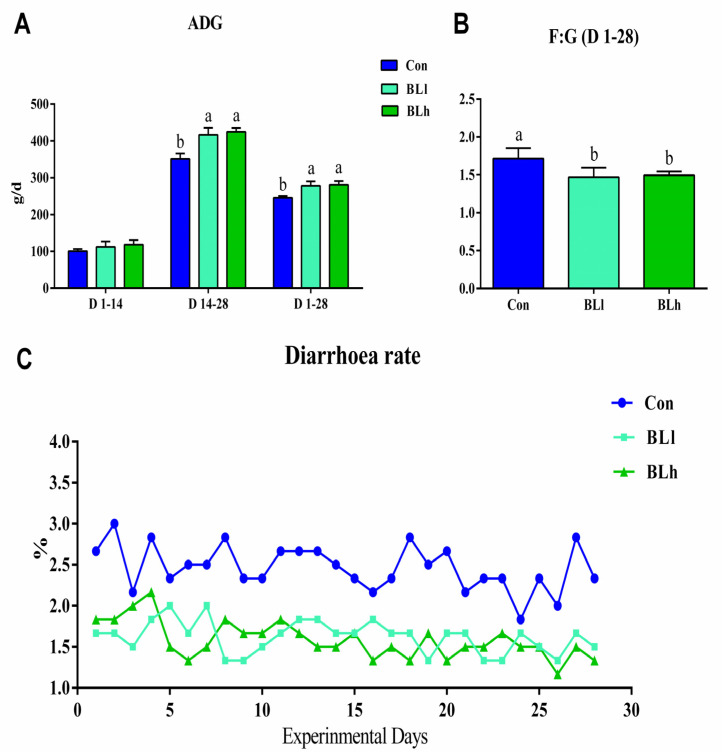
Effects of *B. licheniformis* on the growth performance of piglets. (**A**) ADG, (**B**) F:G, (**C**) Diarrhea index. Con, control; BLl, 10^9^ CFU *Bacillus licheniformis*/kg; BLh, 10^10^ CFU *Bacillus licheniformis*/kg. a, b Means with different superscripts in the same row show significant differences (*p* < 0.05). Note: ADG, average daily gain; F:G, feed: gain. *n* = 6.

Both BLl and BLh treatments significantly increased the ADG of piglets from day 14 to day 28 and from day 1 to day 28, and decreased the F: G ratio compared with the Con treatment (Figure 1A,B); however, no notable difference was observed in the ADG of piglets from day 1 to day 14. Meanwhile, BLl and BLh supplementation decreased the diarrhea index of piglets throughout the study (Figure 1C), with the diarrhea rate of the BLh group being lower than 2%.

### 3.2. Serum Antioxidant Capacity

**Figure 2:**
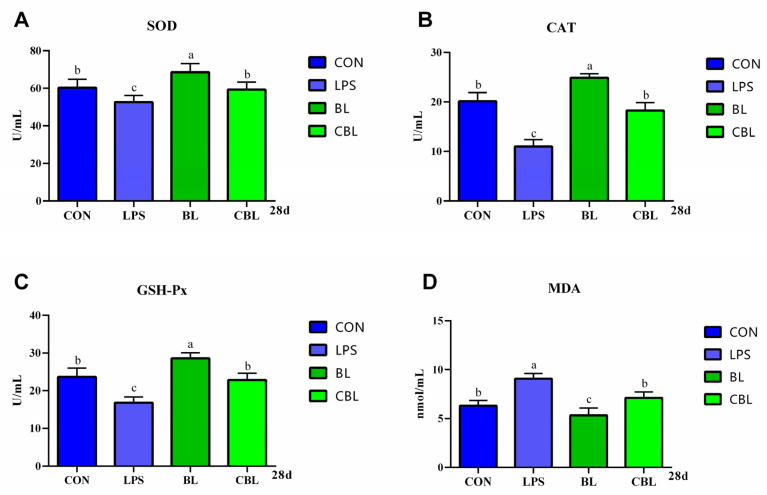
Effects of *B. licheniformis* on serum antioxidant capacity of piglets challenged with LPS. (**A**) CAT, (**B**) MDA, (**C**) GSH-Px, (**D**) SOD. Note: CAT, catalase; MDA, malondialdehyde; GSH-Px, glutathione peroxidase; SOD, superoxide dismutase. Con, control piglets injected with sterilized saline water. LPS, control piglets injected with LPS. BL, 10^10^ CFU *Bacillus licheniformis*/kg piglets injected with sterilized saline water. CBL, 10^10^ CFU *Bacillus licheniformis*/kg piglets injected with LPS. a, b, c Means with different superscripts in the same row show significant differences (*p* < 0.05). *n* = 6.

In comparison to piglets fed the Con diet, BL supplementation significantly increased serum CAT activity in piglets (Figure 2A). Moreover, CBL treatment significantly decreased MDA concentrations compared with the LPS-challenged piglets. Additionally, BL-treated piglets had significantly higher SOD activity compared with LPS-treated piglets, whereas the CBL and control groups showed an increasing trend. There were no significant differences in GSH-Px activity between the treatment groups.

### 3.3. Serum Immune Response

**Figure 3:**
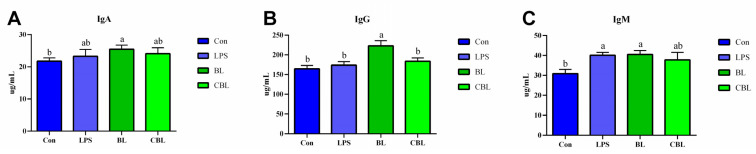
Effects of *B. licheniformis* on serum immunoglobulins of piglets challenged with LPS. (**A**) IgA, (**B**) IgG, (**C**) IgM. CON, control injected with STER; BL, 10^10^ CFU *Bacillus licheniformis*/kg. Note: Con, control piglets injected with sterilized saline water; LPS, control piglets injected with LPS; BL, 10^10^ CFU *Bacillus licheniformis*/kg piglets injected with sterilized saline water; CBL, 10^10^ CFU *Bacillus licheniformis*/kg piglets injected with LPS. a, b Means with different superscripts in the same row show significant differences (*p* < 0.05). *n* = 6.

Compared with the control group, BL-supplemented piglets had significantly higher IgA, IgG, and IgM levels (Figure 3A–C). Compared with LPS treatment, BL supplementation significantly improved the serum concentration of IgG. In addition, piglets in the CBL group showed a trend of increasing IgA and IgG levels in serum compared with piglets in the LPS group.

### 3.4. Ileal Morphology

**Figure 4:**
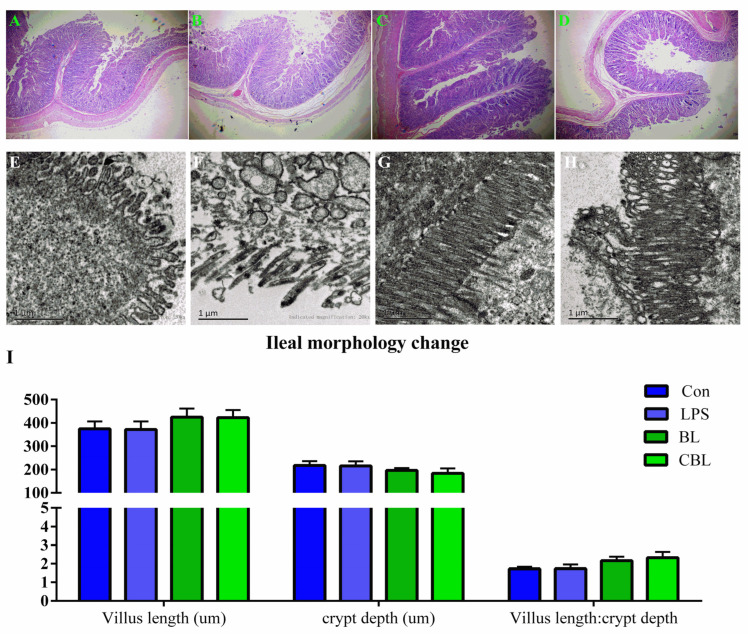
Effects of *B. licheniformis* on ileal morphology of piglets challenged with LPS. (**A**–**D**) HE pictures of ilea of Con, LPS, BL, and CBL piglets; (**E**–**H**) SEM pictures of ilea in Con, LPS, BL, and CBL; (**I**) ileal morphology changes in Con, LPS, BL, and CBL. Note: Con, control piglets injected with sterilized saline water; LPS, control piglets injected with LPS; BL, 10^10^ CFU *Bacillus licheniformis*/kg piglets injected with sterilized saline water; CBL, 10^10^ CFU *Bacillus licheniformis*/kg piglets injected with LPS. *n* = 6.

Hematoxylin and eosin staining and SEM were used to detect changes in the ileal morphology of piglets (Figure 4A–I). More complete ileal villi were observed in both BL and CBL treatment groups than in the control and LPS treatment groups. Although BL and CBL piglets had higher ileal villi, lower crypt depths, and lower villus length: crypt depth ratios compared with Con piglets, no significant differences were found between them.

### 3.5. Colonic SCFAs

**Figure 5:**
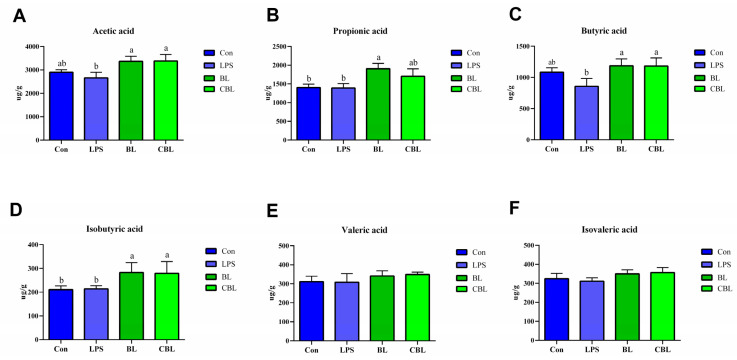
Effects of *B. licheniformis* on colonic SCFAs of piglets challenged with LPS. (**A**) acetic acid, (**B**) propionic acid, (**C**) butyric acid, (**D**) isobutyric acid, (**E**) valeric acid, (**F**) isovaleric acid. Note: Con, control piglets injected with sterilized saline water; LPS, control piglets injected with LPS; BL, 10^10^ CFU *Bacillus licheniformis*/kg piglets injected with sterilized saline water; CBL, 10^10^ CFU *Bacillus licheniformis*/kg piglets injected with LPS. a, b Means with different superscripts in the same row show significant differences (*p* < 0.05). *n* = 6.

Administration of BL significantly increased the concentration of propionic acid and isobutyric acid compared to Con and LPS piglets (Figure 5A–F). The concentrations of acetic, butyric, and isobutyric acids in CBL piglets were significantly higher than in LPS piglets. The BL treatment also increased the concentrations of acetic and butyric acids compared with the Con treatment, although no significant differences were observed between them.

### 3.6. Fecal SCFAs

**Figure 6:**
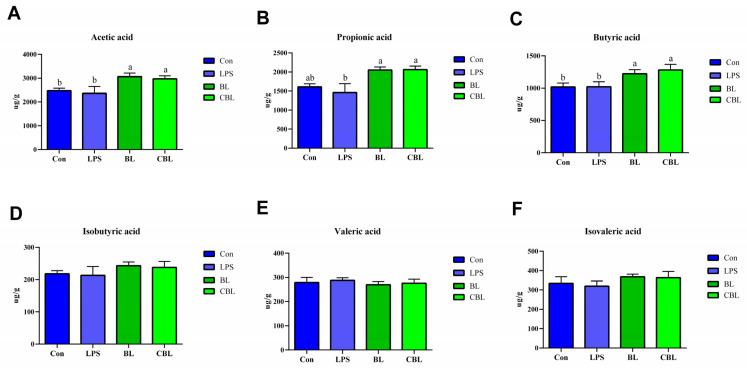
Effects of *B. licheniformis* on fecal SCFAs of piglets challenged with LPS. (**A**) acetic acid, (**B**) propionic acid, (**C**) butyric acid, (**D**) isobutyric acid, (**E**) valeric acid, (**F**) isovaleric acid. Note: Con, control piglets injected with sterilized saline water; LPS, control piglets injected with LPS; BL, 10^10^ CFU *Bacillus licheniformis*/kg piglets injected with sterilized saline water; CBL, 10^10^ CFU *Bacillus licheniformis*/kg piglets injected with LPS. a, b Means with different superscripts in the same row show significant differences (*p* < 0.05). *n* = 6.

The fecal contents of acetic acid and butyric acid were significantly increased by BL and CBL treatments in comparison with the Con and LPS treatments (Figure 6A–F). Both BL and CBL groups had significantly increased acetic acid, propionic acid, and butyric acid levels compared to the LPS group. No significant differences were found in the isobutyric, valeric, and isovaleric acid contents of any of the samples.

### 3.7. Colonic Microbial Metagenome

**Figure 7:**
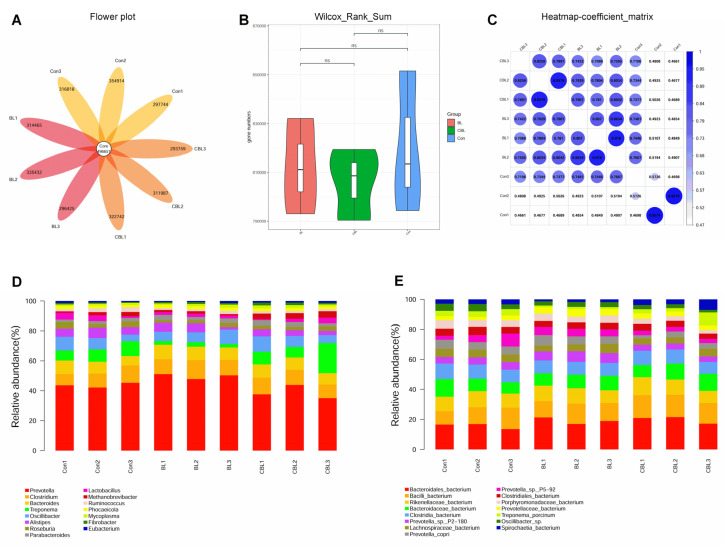
Effects of *B. licheniformis* on colonic microbial metagenome of piglets challenged with LPS. (**A**) flower plot, (**B**) anosim analysis, (**C**) heatmap coefficient matrix, (**D**) relative abundance of the top 15 genera, (**E**) relative abundance of the top 15 species. Note: Con, control piglets injected with sterilized saline water; LPS, control piglets injected with LPS; BL, 10^10^ CFU *Bacillus licheniformis*/kg piglets injected with sterilized saline water; CBL, 10^10^ CFU *Bacillus licheniformis*/kg piglets injected with LPS. *n* = 3.

Metagenome analysis was conducted to investigate the effects of BL on the cecal microbial structure in piglets (Figure 7). The gene flower plot showed that 496,651 core genes were shared by all samples, with each sample having approximately 300,000 genes (Figure 7A). No significant differences were observed in the violin plots of gene numbers between the three treatments (Figure 7B). The heatmap coefficient of the samples in each treatment group was higher than in other treatment groups, indicating that the BL and CBL treatments were different from the Con treatment (Figure 7C). The top 15 genera in all samples were *Prevotella*, *Clostridium*, *Bacteroides*, *Treponema*, *Oscillibacter*, *Alistipes*, *Roseburia*, *Parabacteroides*, *Lactobacillus*, *Methanobrevibacter*, *Ruminococcus*, *Phocaeicola*, *Mycoplasma*, *Fibrobacter*, and *Eubacterium* (Figure 7D). The top eight species in all samples were *Bacteroidales*, *Bacilli*, *Rikenellaceae*, *Bacteroidaceae*, *Clostridia*, *Prevotella* sp. P2-180, *Lachnospiraceae*, and *Prevotella copri* (Figure 7E).

**Figure 8:**
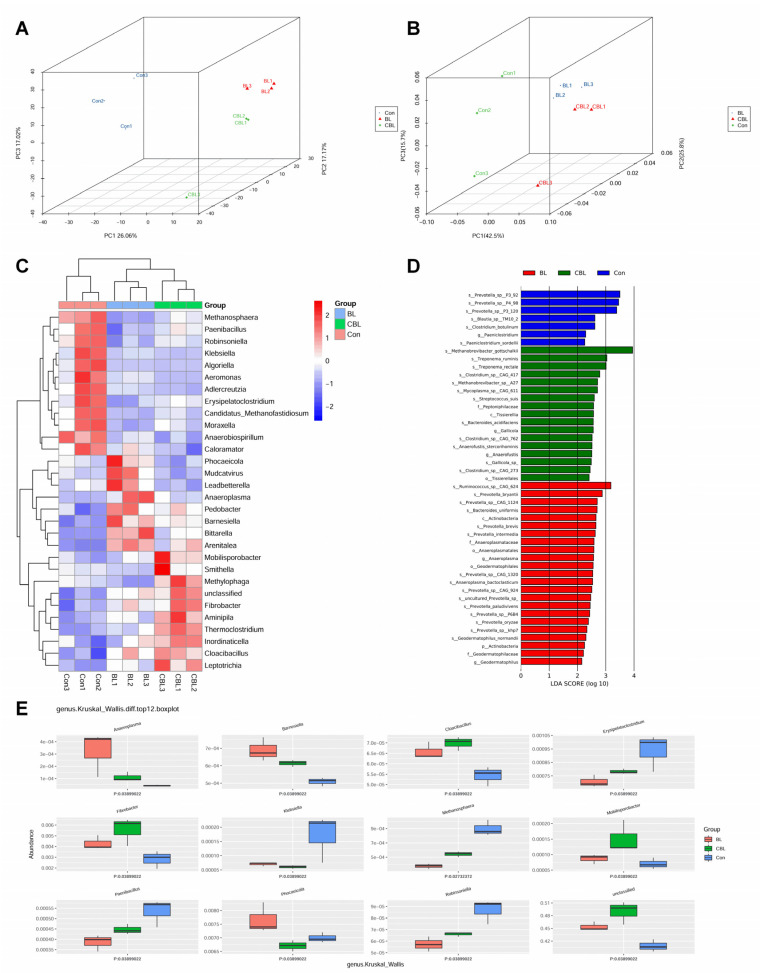
Effects of *B. licheniformis* in colonic microbial microflora of piglets challenged with LPS based on genus level. (**A**) PCA plot, (**B**) PCoA plot, (**C**) heatmap of the top 30 genera, (**D**) LEFSe analysis, (**E**) Kruskal–Wallis analysis. Note: Con, control piglets injected with sterilized saline water; LPS, control piglets injected with LPS; BL, 10^10^ CFU *Bacillus licheniformis*/kg piglets injected with sterilized saline water; CBL, 10^10^ CFU *Bacillus licheniformis*/kg piglets injected with LPS. *n* = 3.

At the genus level, both PCA and PCoA three-dimensional (3D) plots showed that samples from the different treatment groups were well separated, indicating that BL and CBL supplementation dramatically changed the colonic microbial community (Figure 8A,B). The heatmap indicated that the scores for *Methanosphaera*, *Paenibacillus*, *Robinsoniella*, *Klebsiella*, *Algoriella*, *Aeromonas*, *Adlercreutzia*, *Erysipelatoclostridium*, *Candidatus methanofastidiosum*, *Moraxella*, *Anaerobiospirillum*, and *Caloramator* were higher in Con-treated piglets than in the other two treatment groups (Figure 8C). In addition, *Phocaeicola*, *Mudcatvirus*, *Leadbetterella*, *Anaeroplasma*, *Pedobacter*, *Barnesiella*, *Bittarella*, and *Arenitalea* were more abundant in BL-treated piglets, while *Mobilisporobacter*, *Smithella*, *Methylophaga*, unclassified *Fibrobacter*, *Aminipila*, *Thermoclostridium*, *Inordinaticella*, *Cloacibacillus*, and *Leptotrichia* were more abundant in CBL-treated piglets. LEfSe analysis indicated that s__*Prevotella*_sp.__P3_92, s__*Prevotella*_sp.__P4_98, s__*Prevotella*_sp.__P3_120, s__*Blautia*_sp.__TM10_2, s__*Clostridiumbotulinum*, and s_*Paeniclostridium_sordellii* dominated the microflora in Con piglets (Figure 8D). s__*Methanobrevibacter_gottschalkii*, s__*Treponema_ruminis*, s__*Treponema_rectale*, s__*Clostridium*_sp.__CAG_417, s__*Methanobrevibacter*_sp.__A27, s__*Mycoplasma*_sp.__CAG_611, s__*Streptococcus suis*, f__*Peptoniphilaceae*, s__*Bacteroidesacidifaciens*, s__*Anaerofustis_stercorihominis*, s__*Gallicola*_sp., and s__*Clostridium*_sp.__CAG_273 were predominant in the microflora of BL piglets (Figure 8E). The s__*Ruminococcus*_sp.__CAG_624, s__*Prevotella_bryantii*, s__*Prevotella*_sp.__CAG_1124, s__*Bacteroides_uniformis*, s__*Prevotella_brevis*, s__*Prevotella_intermedia*, g__*Anaeroplasma*, s__*Prevotella*_sp.__CAG_1320, s__*Anaeroplasma_bactoclasticum*, s__*Prevotella*_sp.__CAG_924, s__uncultured_*Prevotella*_sp., s__*Prevotella_paludivivens*, s__*Prevotella*_sp.__P6B4, s__*Prevotella*_sp.__khp7, s__*Geodermatophilus_normandii*, and g__*Geodermatophilus* dominated the microflora of CBL piglets. BL supplementation significantly increased the abundance of *Anaeroplasma*, *Barnesiella*, and *Phocaeicola*, and decreased the abundance of *Erysipelatoclostridium*, *Methanosphaera*, *Robinsoniella*, and *Paenibacillus* in the cecum of piglets compared with the Con and LPS groups. In addition, CBL piglets had more *Cloacibacillus*, *Fibrobacter*, and *Mobilisporobacter*, and fewer *Phocaeicola* and *Klebsiella*.

**Figure 9:**
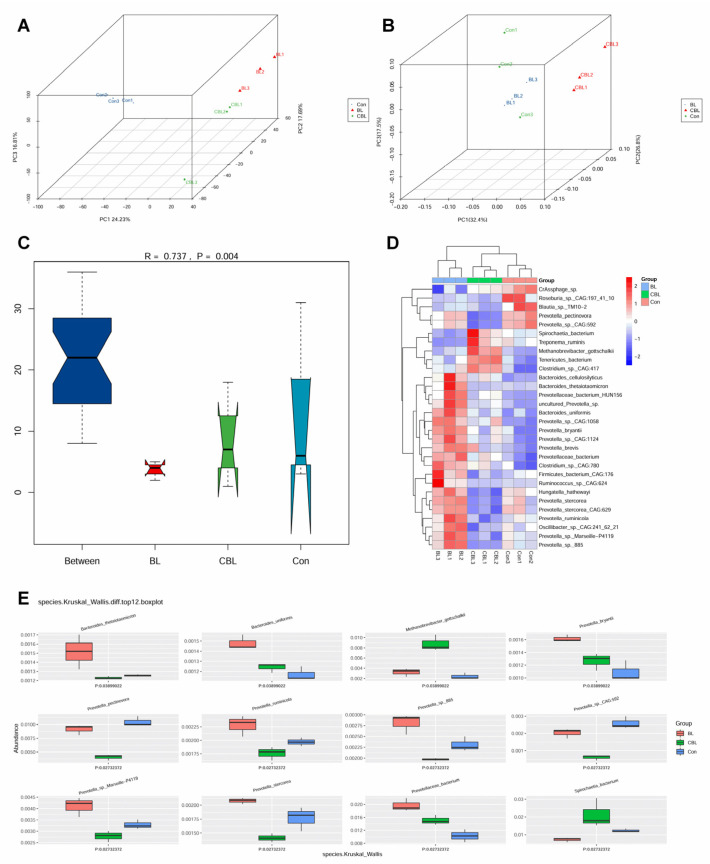
Effects of *B. licheniformis* on colonic microbial microflora of piglets challenged with LPS based on specie level. (**A**) PCA plot, (**B**) PCoA plot, (**C**) Anosim analysis, (**D**) Heatmap of the top 30 species, (**E**) Kruskal–Wallis analysis. Note: Con, control piglets injected with sterilized saline water; LPS, control piglets injected with LPS; BL, 10^10^ CFU *Bacillus licheniformis*/kg piglets injected with sterilized saline water; CBL, 10^10^ CFU *Bacillus licheniformis*/kg piglets injected with LPS. *n* = 3.

At the species level, both PCA and PCoA 3D plots showed that all samples were well-separated according to treatment, which revealed that the different groups had distinct cecal microflora (Figure 9A,B). Anosim analysis (R = 0.737, *p* = 0.004) indicated that the difference between the groups was larger than within the groups. It also showed that both BL and CBL treatments changed the microbial structure (Figure 9C). BL piglets had higher *Alistipes*_sp._CAG:435, *Prevotella copri*, *Prevotella* sp., *Prevotella*_sp._Marseille_P4119, *Prevotella*_sp._P2_180, *Prevotellaceae*, *Parabacteroides distasonis*, *Prevotella*_sp._P5_92, *Porphyromonadaceae*, *Prevotella*_sp._CAG:520, *Ruminococcaceae*, and *Phocaeicola dorei* scores compared with the other two treatment groups. Moreover, Con piglets had higher *Oscillibacter* sp., *Prevotella pectinovora*, and *Treponema bryantii* scores whereas CBL piglets had higher *Bacteroidaceae, Lactobacillus johnsonii, Bacilli, Methanobacteriaceae archaeon, Methanobrevibacter gottschalkii, Oscillibacter*_sp._PC13, *Rikenellaceae, Bacteroidales, Clostridia, Prevotella*_sp._P3-122, *Parabacteroides distasonis*, and *Firmicutes* scores (Figure 9D). The Kruskal–Wallis test was conducted to distinguish the differential microbiota between all treatments (Figure 9E). The abundance of *Bacteroides thetaiotaomicron*, *Bacteroides uniformis*, *Prevotella bryantii*, *Prevotella ruminicola*, *Prevotella* sp.885, *Prevotella* sp.Marseille_P4119, *Prevotella stercorea*, and *Prevotellaceae* were significantly higher in BL piglets than in the other two treatment groups. CBL piglets had a higher abundance of *Methanobrevibacter gottschalkii* and *Spirochaetia*, and fewer *Prevotella pectinovora*, *Prevotella*_sp._CAG:592, and *Prevotella stercorea* compared with the other two treatment groups.

**Figure 10:**
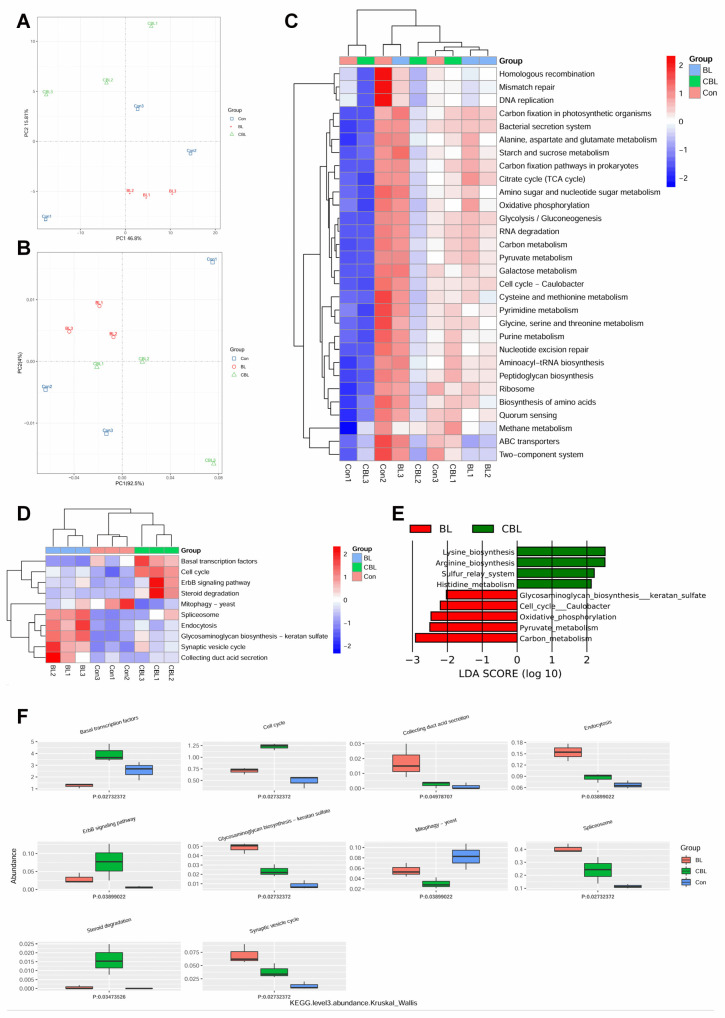
Effects of *B. licheniformis* on the abundance of colonic microbiota genes in piglets challenged with LPS based on the KEGG database. (**A**) PCA plot, (**B**) PCoA plot, (**C**) top 30 metabolic pathways, (**D**) heatmap of the top 10 metabolic pathways on level 2, (**E**) LEFSe analysis, (**F**) Kruskal–Wallis analysis. Note: Con, control piglets injected with sterilized saline water; LPS, control piglets injected with LPS; BL, 10^10^ CFU *Bacillus licheniformis*/kg piglets injected with sterilized saline water; CBL, 10^10^ CFU *Bacillus licheniformis*/kg piglets injected with LPS. *n* = 3.

Based on the KEGG database, the BL and CBL samples were well separated from the Con samples (Figure 10A,B). At level 3, BL-supplemented piglets had higher expression scores for carbon fixation in the photosynthetic bacterial secretion system, carbon fixation pathways in prokaryotes, the citrate cycle (TCA cycle), glycolysis/gluconeogenesis, peptidoglycan biosynthesis, carbon metabolism, pyruvate metabolism, and starch and sucrose metabolism. Lower scores for homologous recombination, mismatch repair, DNA replication, ribosomes, amino acid biosynthesis, and quorum sensing were observed in the CBL piglets (Figure 10C). The heatmap was based on Kruskal–Wallis analysis of the top 30 genera, where basal transcription factors, cell cycle, ErbB signaling pathway, and steroid degradation were higher in the CBL group than in the other two groups (Figure 10D). The BL piglets had higher spliceosome endocytosis, glycosaminoglycan biosynthesis-keratan sulfate, synaptic vesicle cycle, and collecting duct acid secretion scores compared with the other groups. Furthermore, LEfSe analysis indicated that CBL had higher lysine biosynthesis, arginine biosynthesis, sulfur relay system, and histidine metabolism scores (Figure 10E). BL piglets had higher glycosaminoglycan biosynthesis-keratan sulfate, cell cycle Caulobacter, oxidative phosphorylation, pyruvate metabolism, and carbon metabolism scores. Moreover, the meta-analysis indicated that the scores for basal transcription factors, cell cycle, ErbB signaling pathway, and steroid degradation in CBL piglets were significantly higher than for the other two treatment groups. Additionally, the scores for collecting duct acid secretion, endocytosis, glycosaminoglycan biosynthesis-keratan sulfate, spliceosome, and the synaptic vesicle cycle in BL piglets were significantly higher than in the Con and CBL groups (Figure 10F). Interestingly, Con piglets had higher mitophagy scores than BL and CBL piglets.

## 4. Discussion

### 4.1. Effects of BL on the Growth Performance of Weaned Piglets

Weaning is a stressful stage associated with decreased piglet growth and immunity, and induced oxidative damage, gastrointestinal dysfunction, and diarrhea [2]. Previous studies have confirmed the positive effects of multiple *Bacillus species*-based probiotics in improving growth performance and controlling diarrhea in weaning piglets [16,17]. Fermented feed containing *B. licheniformis* and *B. subtilis* dramatically decreases the diarrhea rate and feed intake/body weight gain ratio [18]. *B. licheniformis*-fermented feed can be used as a potential substitute for antibiotics to defend against post-weaning diarrhea during pig production [19]. Zong et al. [20] found that *B. lichenformis* and *C. butyricum* supplementation decreased the incidence of diarrhea in weaning piglets. Similar to the results of the above studies, the 10^10^ CFU *B. lichenformis*/kg treatment significantly increased ADG and decreased the F: G ratio and diarrhea rate in piglets in the current experiment, with the beneficial effects of BLh being better than those of BLl. Combined with our previous study (unpublished data), we chose BLh to investigate the influence of dietary BL on LPS-challenged piglets.

### 4.2. Effects of BL on Serum Antioxidant Capacity of Weaned Piglets Challenged with LPS

The activities of CAT, SOD, GSH-Px, and other associated enzymes represent the host’s antioxidative capacity, in which enzymes are produced to scavenge excess ROS and maintain homeostasis [21]. MDA is involved in lipid oxidation in the host and is caused by lipid peroxidation [22]. LPS treatment decreased serum CAT activity and increased MDA concentration, indicating that the LPS challenge induced oxidative damage in the present study. In a previous study, *B. licheniformis* supplementation at a dose of 5 × 10^8^ CFU/kg was confirmed to increase serum T-AOC, SOD, and GSH-Px activities, and to decrease MDA levels in piglets [13]. Dietary supplementation with a *Bacillus* mixture reduced the harmful effects of oxidative injury in poultry [23]. It has been confirmed that a mixture of *B. licheniformis* and *S. cerevisiae* treatment leads to higher activity of serum SOD and GSH-Px in fattening lambs [24]. Similarly, BL supplementation improved the oxidative capacity of weaned piglets by increasing serum CAT activity and decreasing serum MDA concentrations in the present study.

### 4.3. Effects of BL on Serum Immunoglobulins of Weaned Piglets Challenged with LPS

Immunoglobulins are critical elements in the immune systems of animals and are mainly present in the serum [25]. It was found that oral administration of *B. subtilis* significantly boosted the contents of serum IgG and ileum IgA antibodies in piglets challenged with porcine epidemic diarrhea virus [26]. In another study, *B. licheniformis* supplementation significantly improved serum IgA and IgM levels in piglets [13]. One study conducted by Zong et al. [20] found that dietary *B. licheniformis* and *C. butyricum* induced higher serum IgG and IgA concentrations in piglets. Our previous study confirmed that piglets fed with probiotic compounds containing BL, *C. butyricum*, and *B. subtilis* had higher levels of serum IL-6, TNF-α, and IL-1β [2]. Consistent with the above studies, dietary BL treatment enhanced the immune response of piglets by increasing serum IgA, IgM, and IgG levels.

### 4.4. Effects of BL on Ileal Morphology of Weaned Piglets Challenged with LPS

As a protective barrier, the intestinal epithelium plays a vital role in nutrient absorption, and its morphology is a key indicator of intestinal development and function in weaned piglets [2]. Chen et al. (2020) found that an LPS challenge decreased intestinal villus height and increased crypt depth [8]. One previous study showed that BL supplementation combined with *C. butyricum* and *B. subtilis* played a positive role in maintaining intestinal morphology, including enhancing ileal villus integrity and alleviating jejunal and ileal apoptosis [2]. It is reported that supplementation with *B. licheniformis* DSM5749 optimized intestinal morphological integrity and enhanced tight junctions in laying hens [4]. In the present study, we found that dietary BL numerically improved ileal villus length and decreased crypt depth, although there were no significant differences. HE and SEM images showed a more complete ileal villus morphology in BL-supplemented piglets. Sun et al. [27] also supported results similar to our data, showing that *B. licheniformis* enhanced intestinal mucosal integrity, as evidenced by an increased villus height and villus height to crypt depth ratio, and induced higher jejunal mucosal mRNA and occludin contents.

### 4.5. Effects of BL on the Colonic and Fecal SCFAs of Weaned Piglets Challenged with LPS

Numerous studies have indicated that intestinal-derived SCFAs not only provide the major energy for the growth of colonocytes but also modulate the immune and inflammatory responses in the host, while simultaneously regulating the microbial community [28]. Additionally, SCFAs fermented with indigestible carbohydrates positively affect the growth performance of newborn mice [29,30,31] and butyrate induced cell growth by providing energy to colonic epithelial cells and regulating host immune responses. Probiotic treatment led to a higher SCFAs content, which could be beneficial to the piglets’ intestinal environment [32]. Probiotic supplementation results in a higher proportion of SCFAs, mainly acetic acid, which may reduce post-weaning diarrhea in piglets [32]. Direct feeding on *B. subtilis* and *B. licheniformis* also induces an increase in ruminal isovalerate and isobutyrate levels in cows [33]. Similarly, our study showed that BL supplementation increased colonic and fecal acetic and butyric acid concentrations.

### 4.6. Effects of BL on the Colonic Microbiota of Weaned Piglets Challenged with LPS

The intestinal microflora plays an important role in taking advantage of nutrients, manufacturing short-chain fatty acids, enhancing immune responses, and developing resistance against harmful bacteria [19]. It is well known that antibiotics and probiotics can be used as feed additives to modulate gut microbial diversity and composition [34]. A compound containing *B. licheniformis* was confirmed to alter piglets’ intestinal microbial diversity [35]. A recent study conducted by Jiao et al. [36] confirmed that dietary *Bacillus* spp. combined with MCFA enhances the intestinal barrier by altering the microbiota in piglets. Dietary combinations of *B. licheniformis* and *B. subtilis* reshaped the intestinal microbiota of weaned piglets challenged with enterotoxigenic *E. coli*. [37]. Both PCA and PCoA showed that BL supplementation modulated the colonic microbiota of LPS-challenged piglets. Combined with LEfSe and meta-analysis, *Prevotella* species were found to be the predominant bacteria in the BL-treated piglets. It is well known that *Prevotella* species have capacities for degrading polysaccharides through digestive enzymes, which promote the production of SCFAs in the intestines of growing pigs [38]. Moreover, *Prevotella* species can enhance feed intake and nutrient availability in piglets [39]. These results are consistent with our findings. Additionally, the abundance of *Proteobacteria* (including *Acinetobacter*, *Stenotrophomonas*, *Brevundimonas*, and *Herbaspirillum*) and *Anaerostipes* was negatively associated with isovalerate content [40], which also supported our previous SCFAs data.

The heatmap and meta-statistics showed that BL-treated piglets had higher spliceosome endocytosis, glycosaminoglycan biosynthesis-keratan sulfate, synaptic vesicle cycle, and collecting duct acid secretion scores. LEfSe analysis indicated that BL piglets had higher glycosaminoglycan biosynthesis keratan sulfate, cell cycle Caulobacter, oxidative phosphorylation, pyruvate metabolism, and carbon metabolism scores, whereas CBL piglets had higher lysine biosynthesis, arginine biosynthesis, sulfur relay system, and histidine metabolism scores. Keratan sulfate is an important cell regulatory factor in the epithelial and mesenchymal tissues of hosts [41]. It has been reported that oxidative phosphorylation plays a key role in the mitochondrial electron transport chain by generating cellular ATP [42]. Pyruvate produced by glycolysis produces two adenosine triphosphate and two nicotinamide adenine dinucleotide molecules per glucose molecule [43]. Dietary arginine is typically used to relieve various conditions, including LPS-induced oxidative stress, and is also a leader in metabolically active substances [44]. Hence, microbial modulation by BL supplementation influenced the metabolism of piglets challenged with LPS.

## 5. Conclusions

The present study showed that *B. licheniformis* supplementation at 10^10^ cfu/Kg dosage improved growth performance by increasing ADG and decreasing F: G, enhanced serum antioxidant capacity by increasing catalase activity, increased colonic SCFAs contents and modulated the colonic microbiota of weaning piglets challenged with LPS.

## Figures and Tables

**Table 1:** Composition of the basal diet (air-dry basis, %).

Ingredients	Content	Nutrient Level	Content
Corn	55.00	DE, MJ/kg	14.17
Wheat middling	3.50	CP, %	20.35
Phospholipid	2.00	Lys, %	1.34
Whey powder	5.00	Met + Cys, %	0.77
Extruded soybean	7.30	Thr, %	0.80
Soybean meal	18.50	Ca, %	0.95
Fish meal	5.00	TP, %	0.65
Dicalcium phosphate	1.00	AP, %	0.48
Limestone	1.10		
NaCl	0.10		
L-Lysine HCl	0.35		
DL-methionine	0.15		
Premix ^1^	1.00		
Total	100.00		

^1^ Supplied the following per kg of diet: vitamin A, 10,000 IU; vitamin D3, 400 IU; vitamin E, 10 mg; pantothenic acid, 15 mg; vitamin B6, 2 mg; biotin, 0.3 mg; folic acid, 3 mg; vitamin B12, 0.009 mg; ascorbic acid, 40 mg; Fe, 150 mg; Cu, 130 mg; Mn, 60 mg; Zn, 120 mg; I, 0.3 mg; Se, 0.25 mg.

## Data Availability

Data presented are original and not inappropriately selected, manipulated, enhanced, or fabricated.
